# Supplementary material for: Speciation and hydration forces in sodium carbonate/bicarbonate aqueous solutions nanoconfined between mica sheets
Source: Faraday Discuss. 2026 Jan 26. Online ahead of print. doi: 10.1039/d5fd00145e (PMC13130180; doi:10.1039/d5fd00145e)
Supplement: FD-OLF-D5FD00145E-s001 [file FD-OLF-D5FD00145E-s001.pdf]

Supplementary Information (SI) for Faraday Discussions.
This journal is © The Royal Society of Chemistry 2026

Supplementary Information

Speciation and hydration forces in sodium carbonate/bicarbonate aqueous solutions nanoconfined between mica sheets

Daria Turculet, Shurui Miao, Kieran J. Agg and Susan Perkin*

Physical and Theoretical Chemistry Laboratory, Department of Chemistry, University of Oxford, Oxford OX1 3QZ, UK. E-mail: susan.perkin@chem.ox.ac.uk.

Additional Force Profiles

A series of supplementary figures (Figures S1-S20) present the full set of force-distance measurements obtained in the three electrolyte solutions studied: 1 mM $NaHCO_3$, 10 mM $NaHCO_3$, and 10 mM Na_2CO_3. For each solution, multiple independent approach-retraction cycles were recorded to verify reproducibility. The dataset consists of 4 force profiles for 1 mM $NaHCO_3$, and 8 force profiles each for 10 mM $NaHCO_3$ and 10 mM Na_2CO_3. Each supplementary figure is structured in three panels:

- a) Full interaction curve: a zoomed-out force-distance profile showing both approach and retraction curves over the entire measured distance range
- b) Layer-resolved region: a zoomed-in view of the short-range region, illustrating the reproducible, discrete layering features observed on both approach and retraction
- c) Charge-regulation DLVO fit: a log-scale plot of the interaction force versus separation for the approach curve, overlaid with a charge-regulation DLVO fit. This fit yield estimates of the screening length, the effective surface potential (ψ_{eff}) of mica, and the charge-regulation parameter p.

The supplementary table S1 compiles the fit parameters extracted from the charge-regulation DLVO analysis for all force profiles. For each solution, the following values are reported:

- Charge-regulation parameter (p)
- Screening length (κ^{-1})
- Effective surface potential (ψ_{eff})

For each parameter, mean values, standard deviations, and standard errors are calculated across all measurements for that electrolyte solution. These statistical summaries provide a quantitative assessment of the reproducibility and variability of the interfacial properties under the different solution conditions.

Table S1. DLVO parameters for all electrolyte conditions. Mean values, standard deviations (SD), and standard errors (SE) are shown. N denotes the number of force profiles.

**Parameter**	**1 mM $NaHCO_3$** ($N = 4$)			**10 mM $NaHCO_3$** ($N = 8$)			**10 mM Na_2CO_3** ($N = 8$)		
	Mean	SD	SE	Mean	SD	SE	Mean	SD	SE
p	0.874	0.082	0.041	0.740	0.085	0.030	0.800	0.087	0.031
κ^{-1} (nm)	10.405	1.050	0.525	3.291	0.210	0.074	2.327	0.077	0.027
ψ_{eff} (mV)	101.322	10.228	5.114	63.130	3.118	1.102	37.328	7.167	2.534

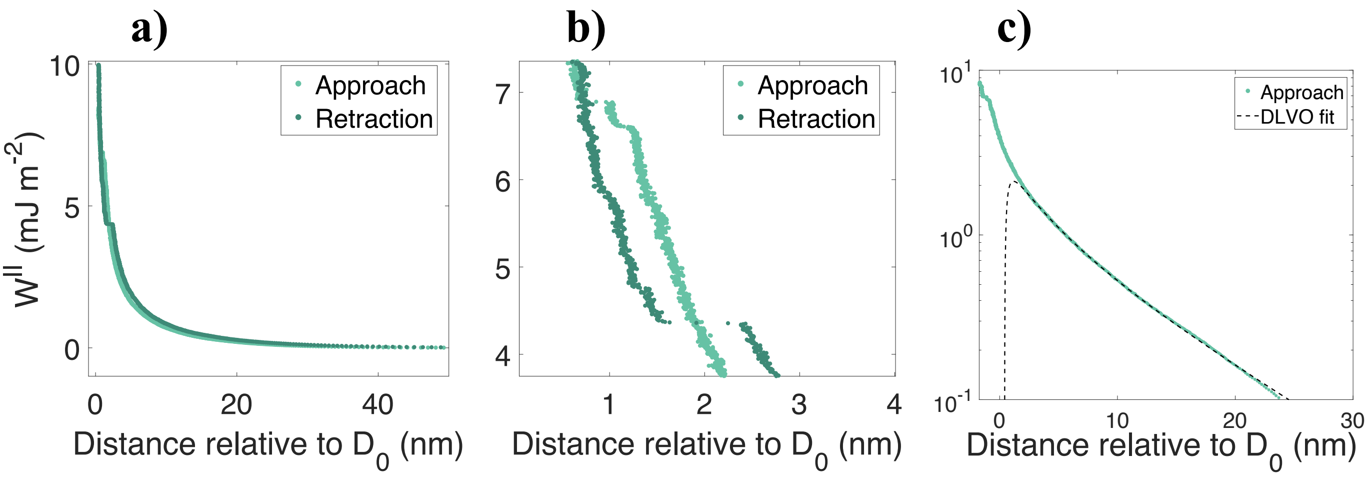

Figure S1. 1 mM $NaHCO_3$ Run 1

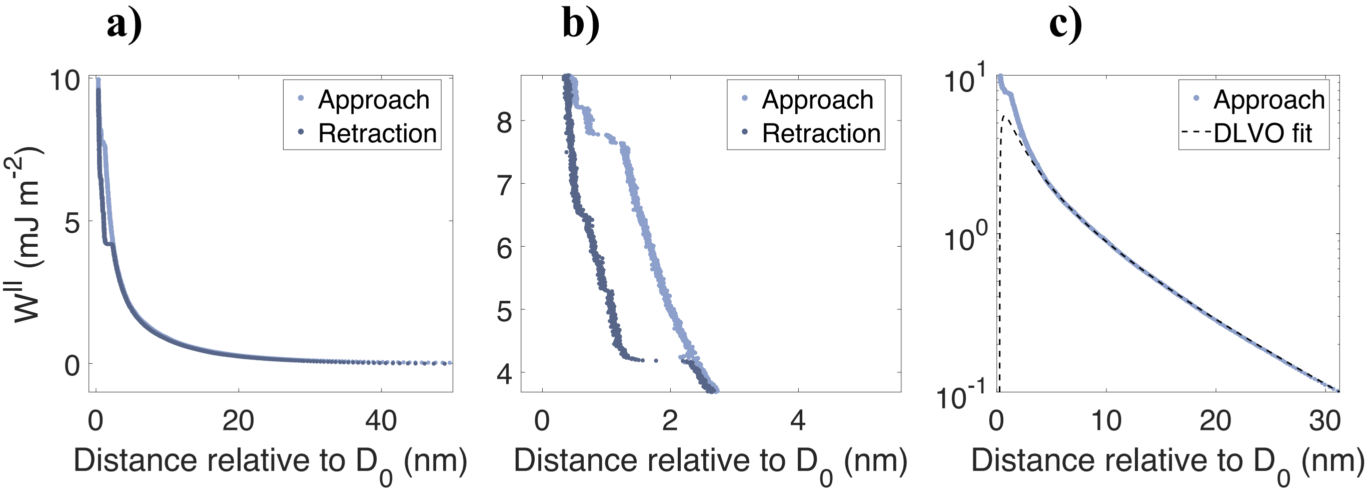

Figure S2. 1 mM $NaHCO_3$ Run 2

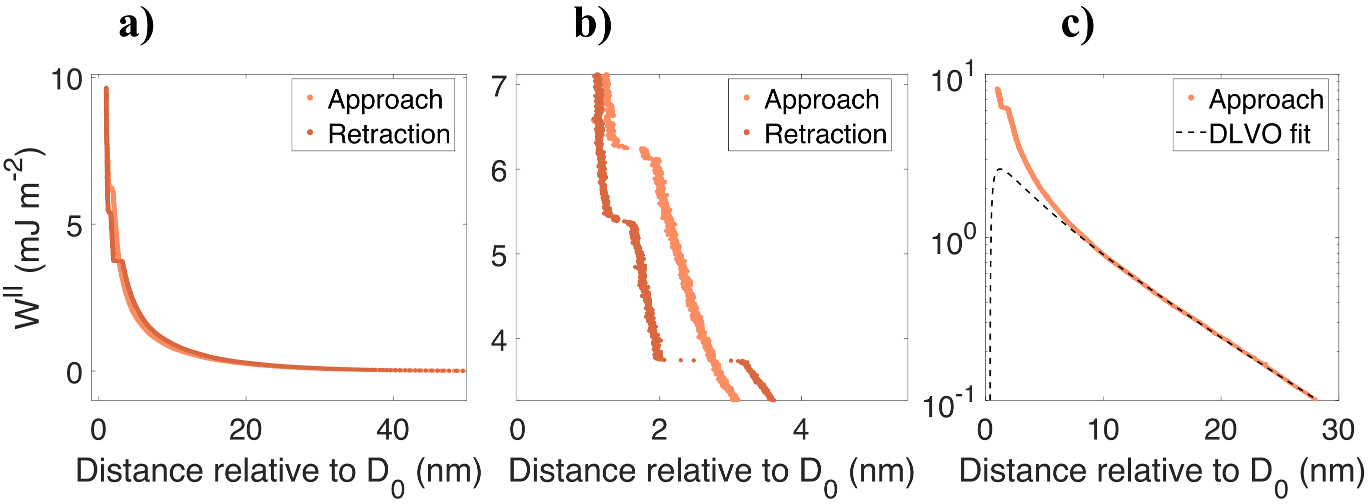

Figure S3. 1 mM $NaHCO_3$ Run 3

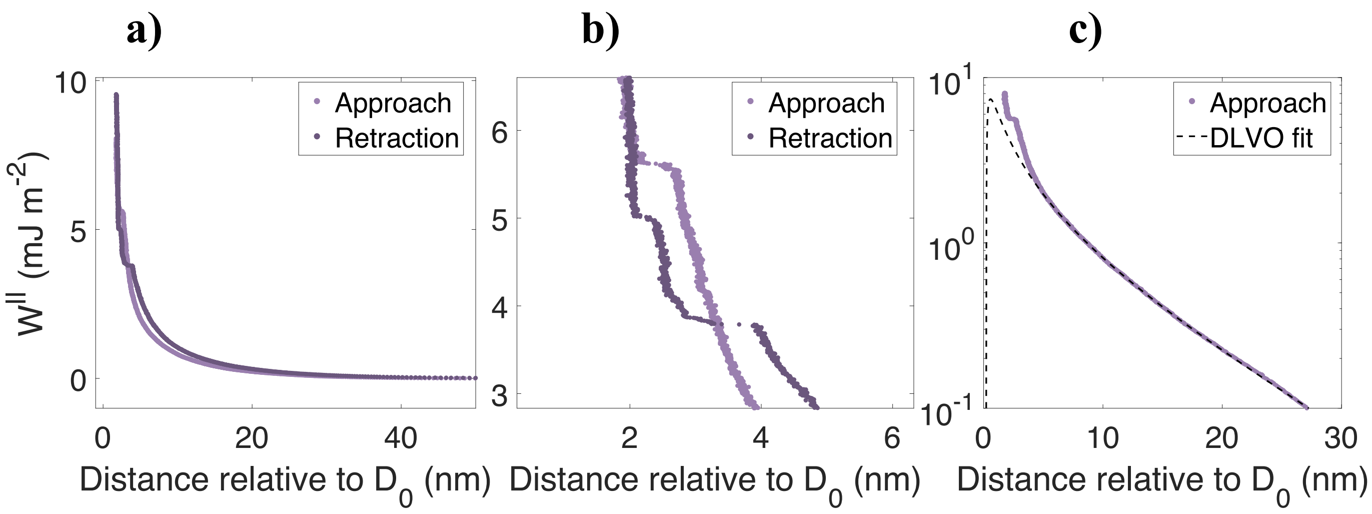

Figure S4. 1 mM $NaHCO_3$ Run 4

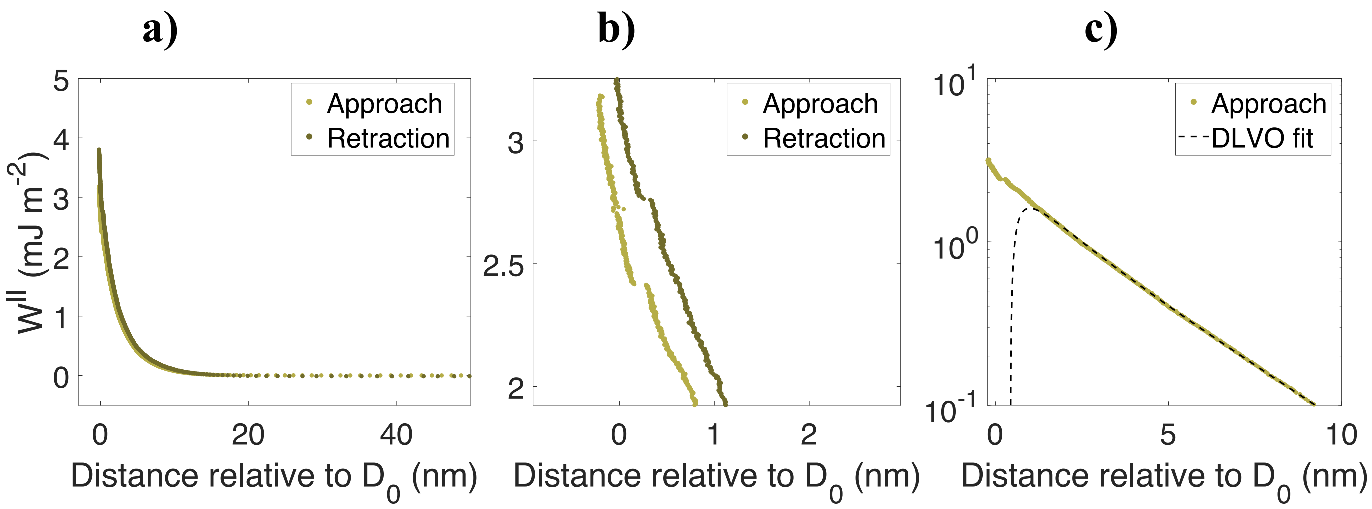

Figure S5. 10 mM $NaHCO_3$ Run 1

Figures/SI Figure 6.pdf

Figure S6. 10 mM $NaHCO_3$ Run 2

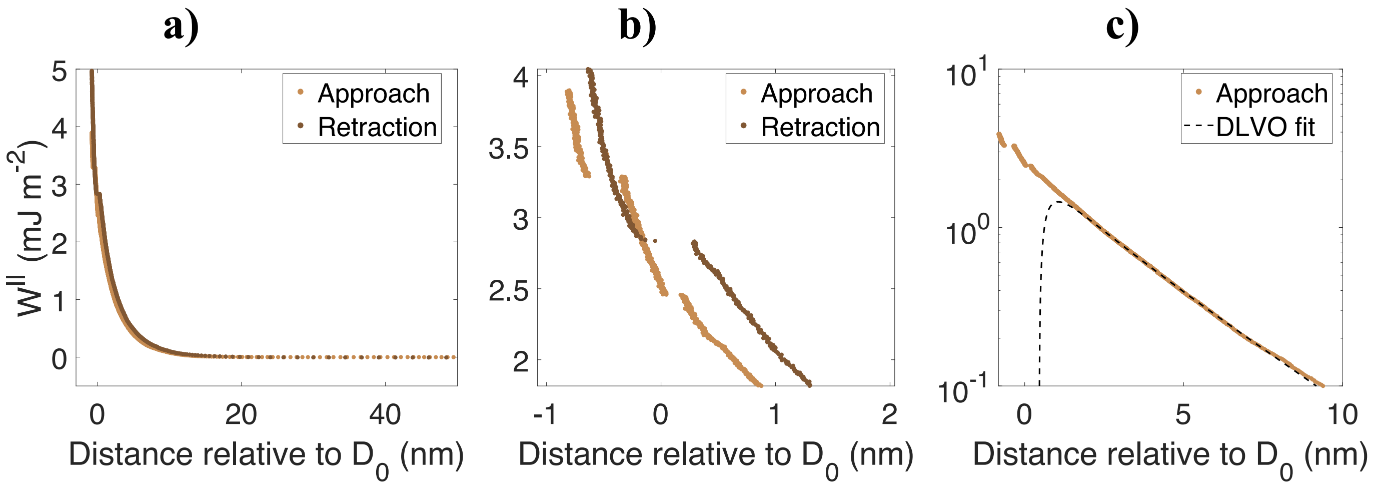

Figure S7. 10 mM $NaHCO_3$ Run 3

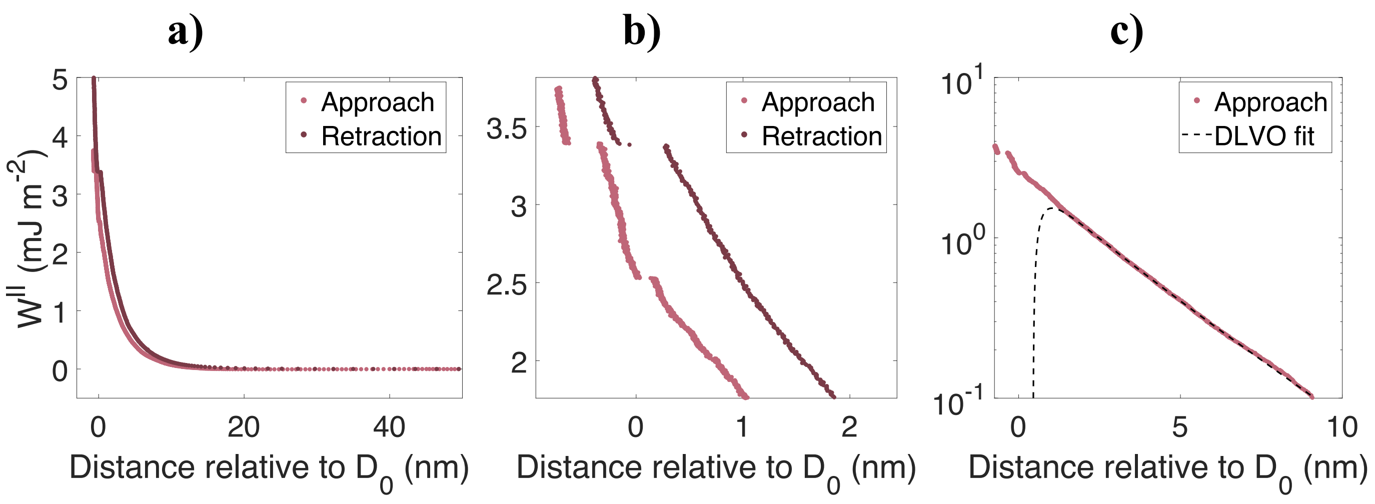

Figure S8. 10 mM $NaHCO_3$ Run 4

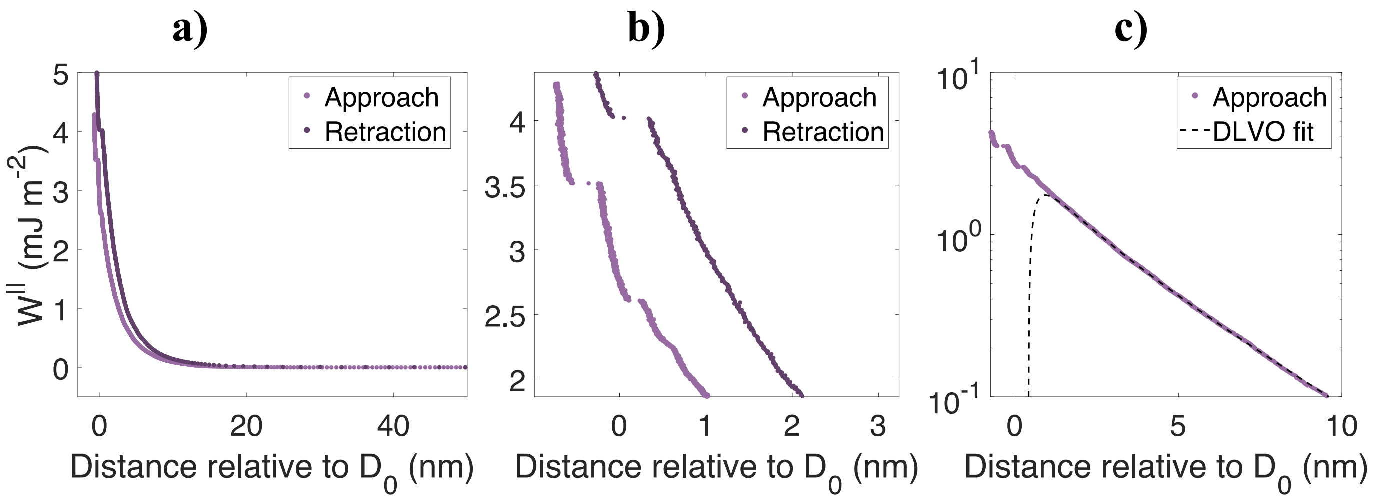

Figure S9. 10 mM $NaHCO_3$ Run 5

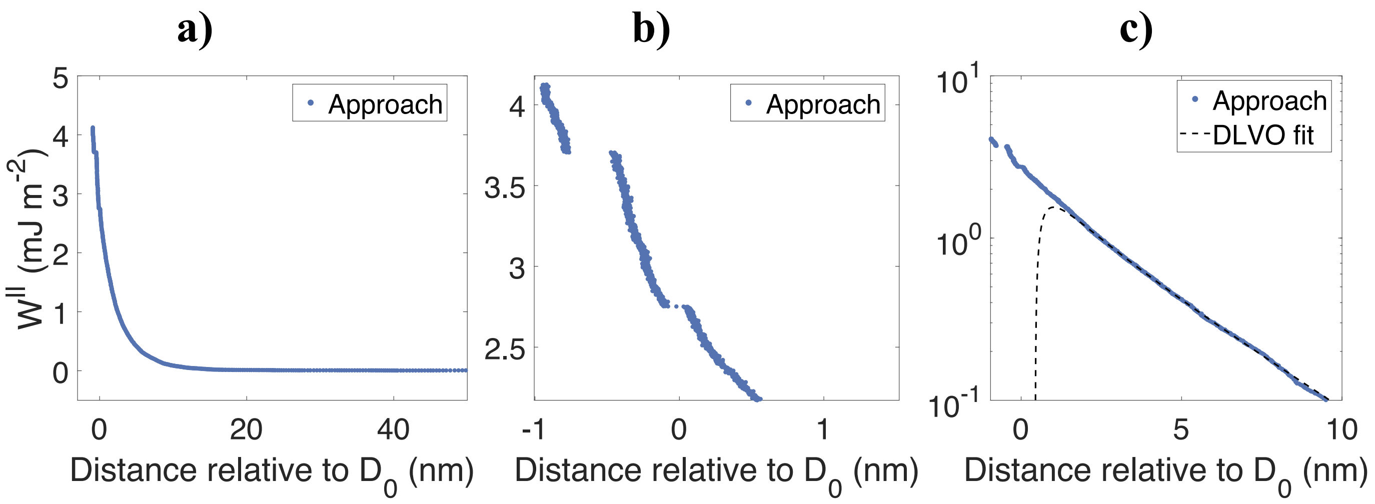

Figure S10. 10 mM $NaHCO_3$ Run 6

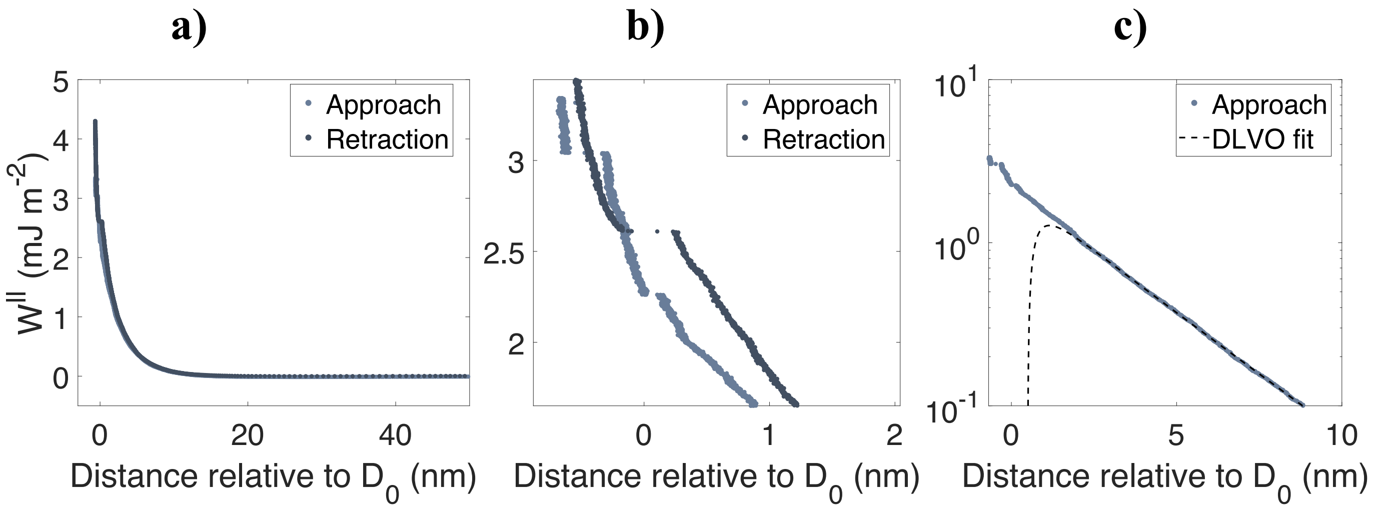

Figure S11. 10 mM $NaHCO_3$ Run 7

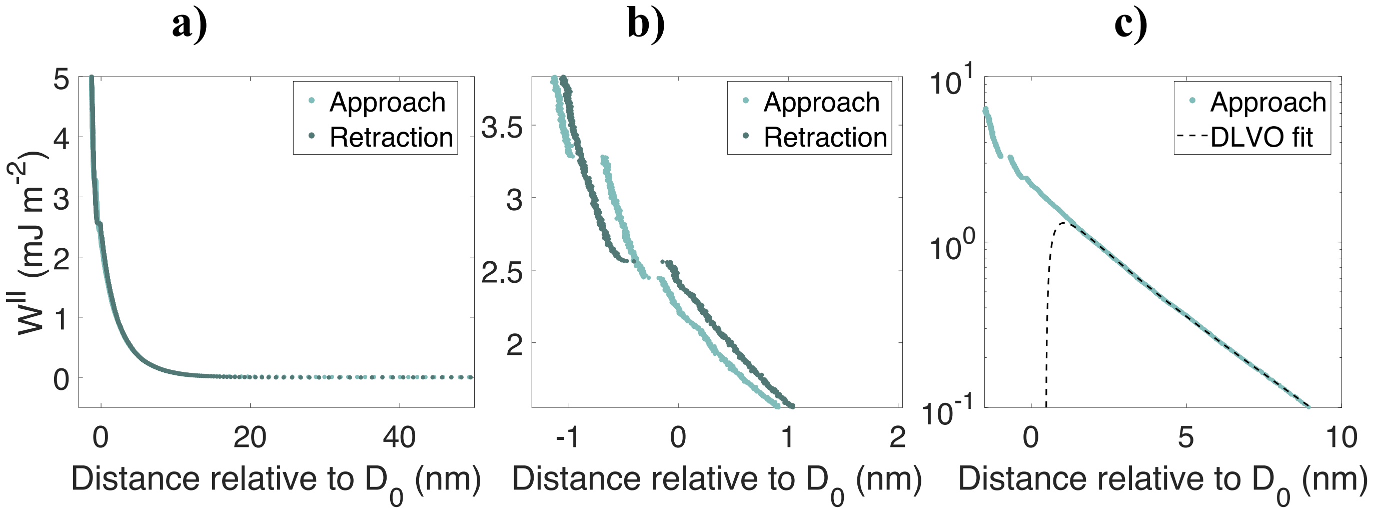

Figure S12. 10 mM $NaHCO_3$ Run 8

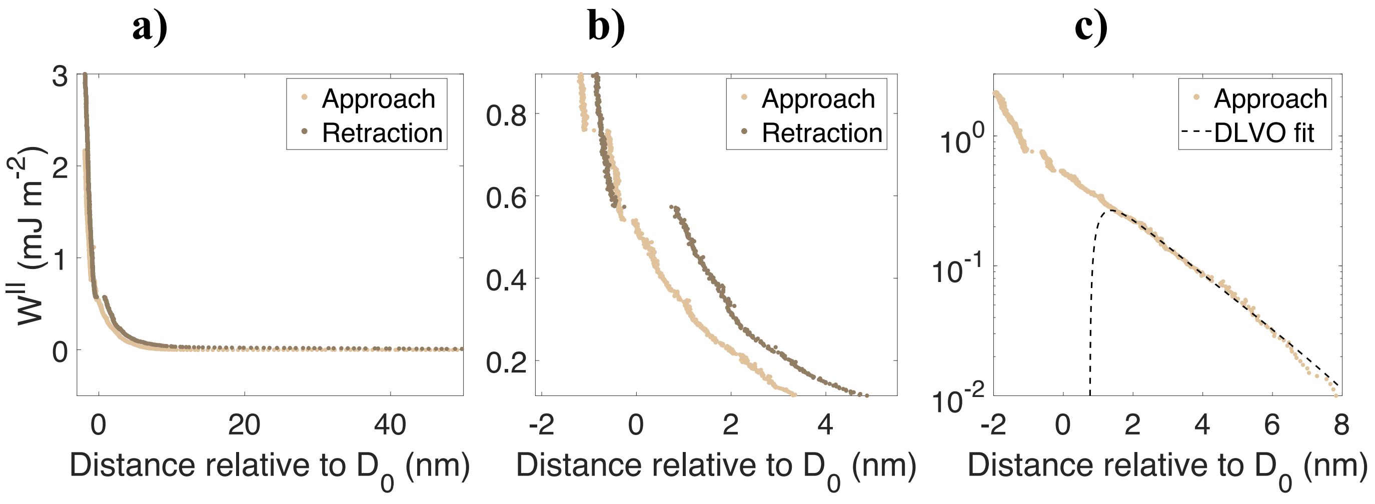

Figure S13. 10 mM Na_2CO_3 Run 1

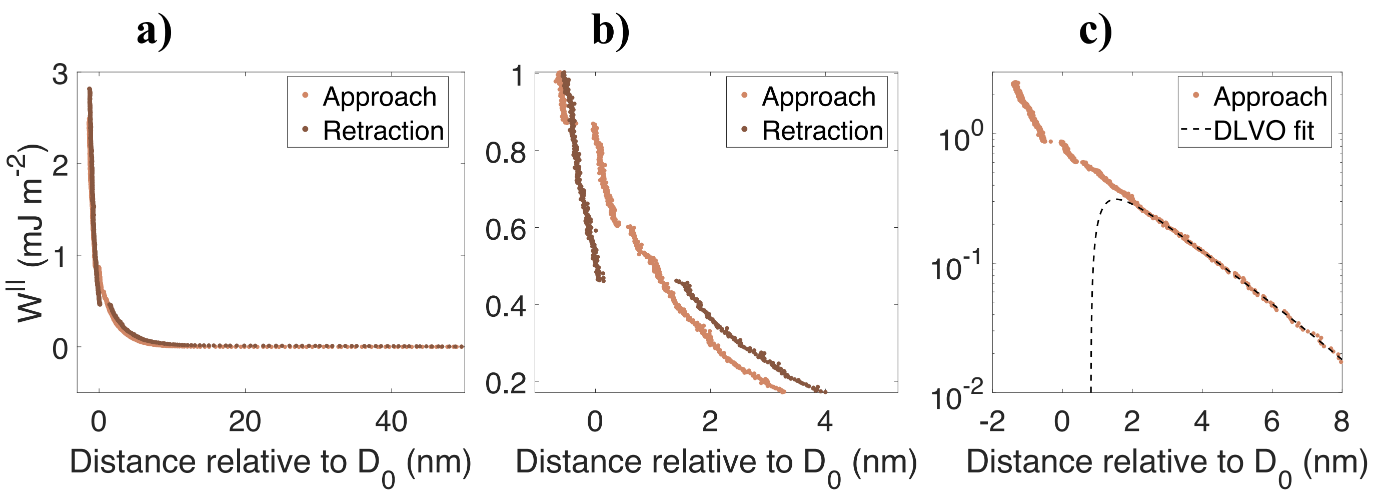

Figure S14. 10 mM Na_2CO_3 Run 2

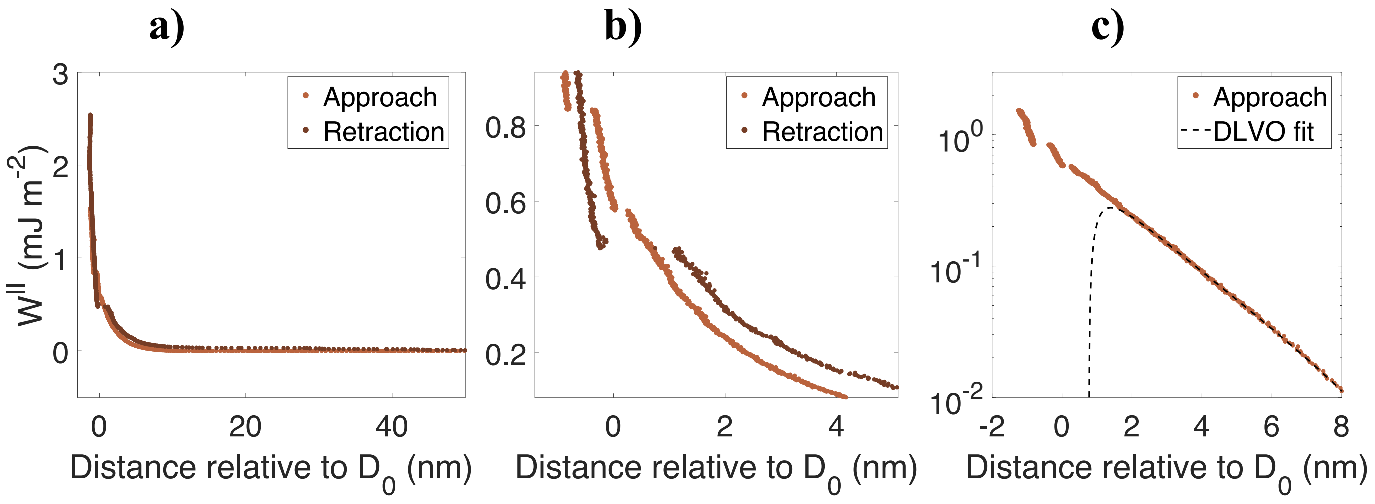

Figure S15. 10 mM Na_2CO_3 Run 3

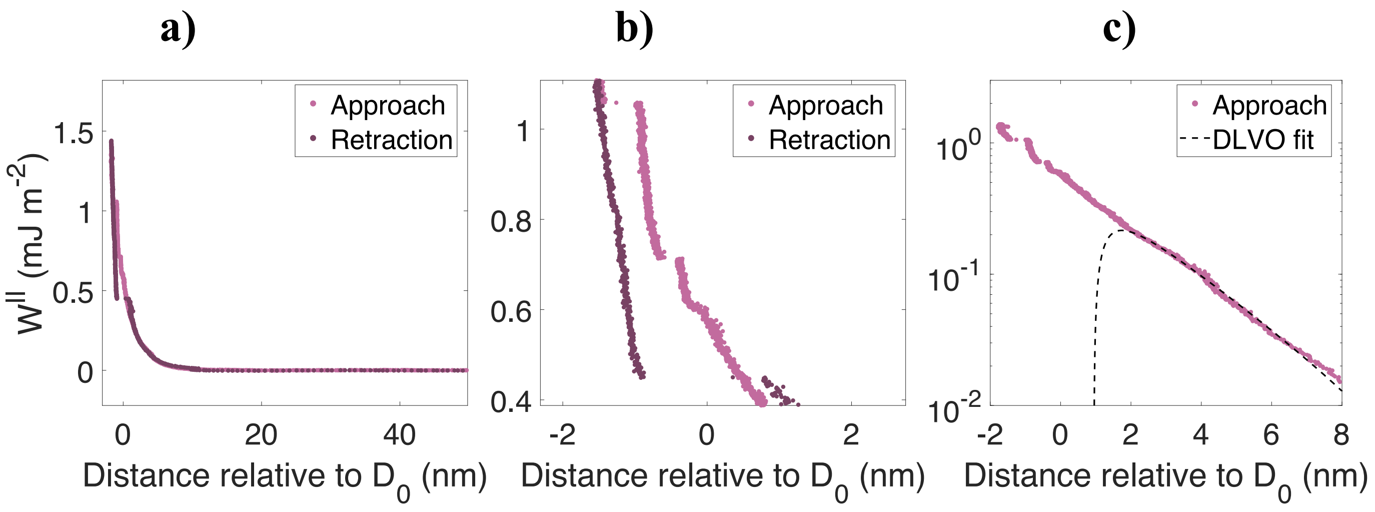

Figure S16. 10 mM Na_2CO_3 Run 4

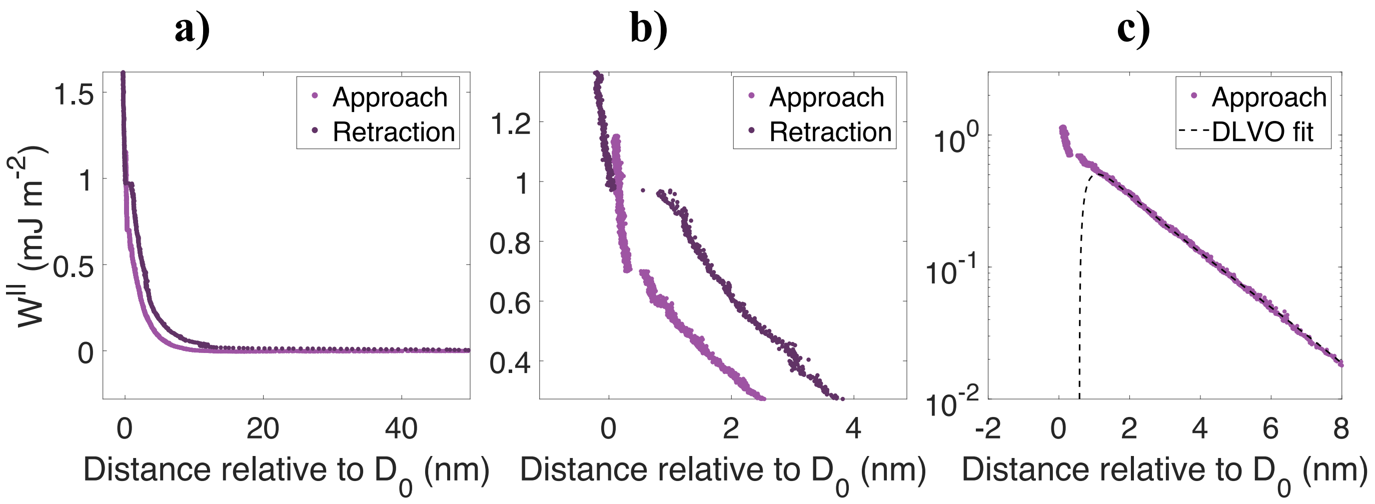

Figure S17. 10 mM Na_2CO_3 Run 5

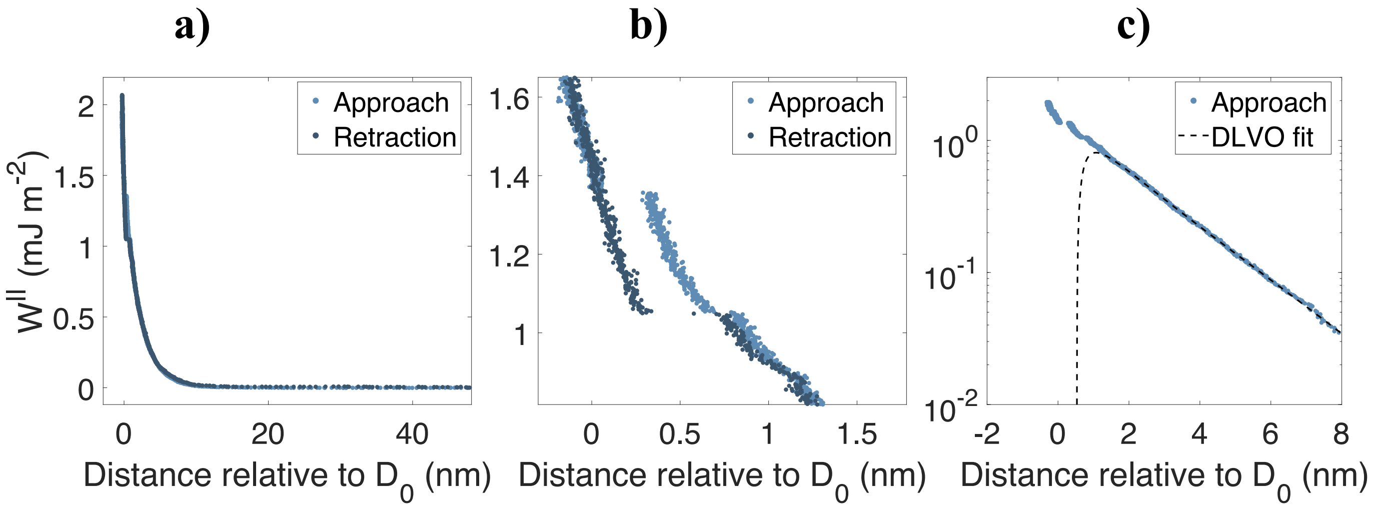

Figure S18. 10 mM Na_2CO_3 Run 6

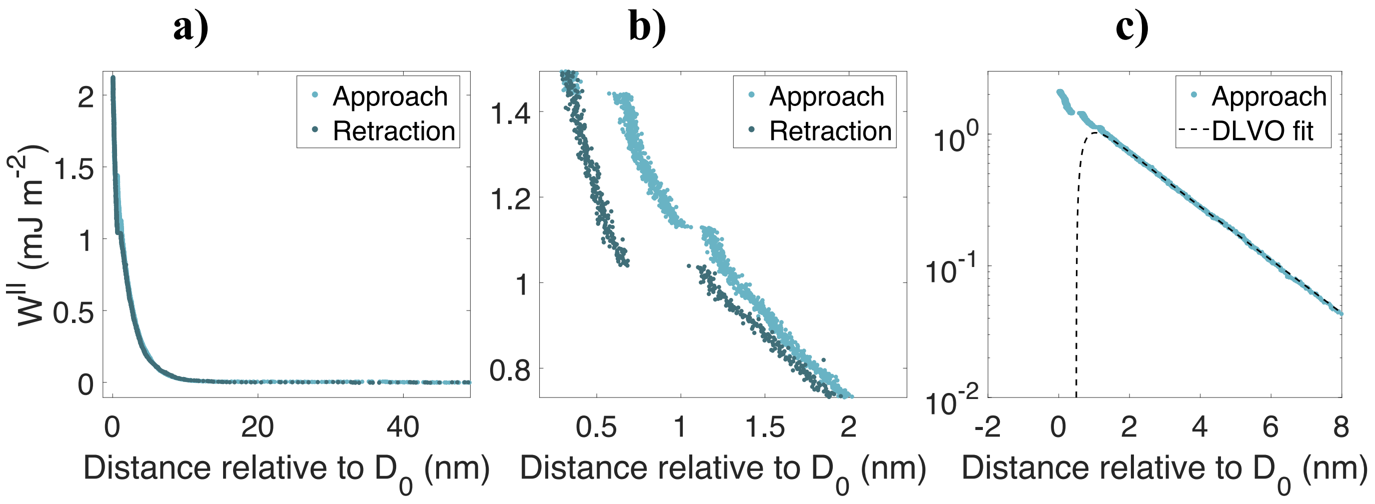

Figure S19. 10 mM Na_2CO_3 Run 7

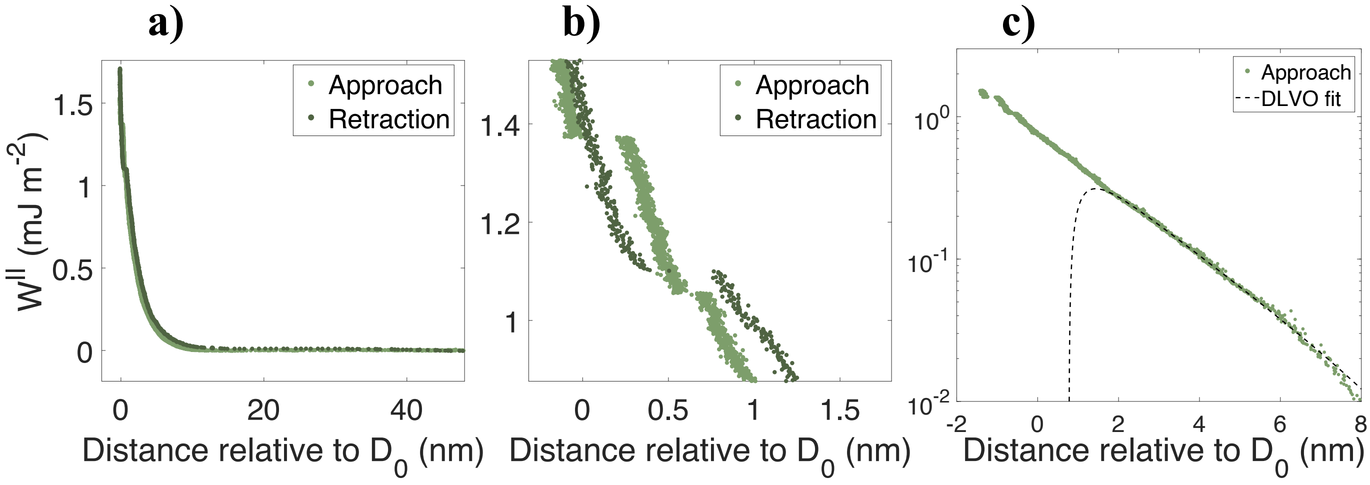

Figure S20. 10 mM Na_2CO_3 Run 8

Derivation of species fractions for the carbonate system

In the following we assume unit activity, such that $\mathrm{pH} = -\log_{10} a_{H^+} = -\log_{10}(\gamma_{H^+} c_{H^+}/c^\circ) = -\log_{10}[\mathrm{H^+}]$, where the notation [X] implies normalised dimensionless concentration, i.e. a 1mM H^+ aqueous solution is written $[\mathrm{H^+}] = 0.001$.

Consider the equilibria

$$\mathrm{H_2CO_3} \rightleftharpoons \mathrm{H^+} + \mathrm{HCO_3}^-, \qquad K_{a1} = \frac{[\mathrm{H^+}][\mathrm{HCO_3^-}]}{[\mathrm{H_2CO_3}]},$$

$$\mathrm{HCO_3}^- \rightleftharpoons \mathrm{H^+} + \mathrm{CO_3}^{2-}, \qquad K_{a2} = \frac{[\mathrm{H^+}][\mathrm{CO_3^{2-}}]}{[\mathrm{HCO_3^-}]}.$$

Total inorganic carbon is

$$C_T = [\mathrm{H_2CO_3}] + [\mathrm{HCO_3^-}] + [\mathrm{CO_3^{2-}}].$$

From the equilibrium constants:

$$[\mathrm{HCO_3^-}] = \frac{K_{a1}[\mathrm{H_2CO_3}]}{[\mathrm{H^+}]}, \qquad [\mathrm{CO_3^{2-}}] = \frac{K_{a1}K_{a2}[\mathrm{H_2CO_3}]}{[\mathrm{H^+}]^2}.$$

Thus

$$C_T = [\mathrm{H_2CO_3}]\left(1 + \frac{K_{a1}}{[\mathrm{H^+}]} + \frac{K_{a1}K_{a2}}{[\mathrm{H^+}]^2}\right).$$

The species fractions f_i are therefore

$$f_0 = \frac{[\mathrm{H_2CO_3}]}{C_T} = \frac{1}{1 + \frac{K_{a1}}{[\mathrm{H^+}]} + \frac{K_{a1}K_{a2}}{[\mathrm{H^+}]^2}},$$

$$f_1 = \frac{[\mathrm{HCO_3^-}]}{C_T} = \frac{\frac{K_{a1}}{[\mathrm{H^+}]}}{1 + \frac{K_{a1}}{[\mathrm{H^+}]} + \frac{K_{a1}K_{a2}}{[\mathrm{H^+}]^2}},$$

$$f_2 = \frac{[\mathrm{CO_3^{2-}}]}{C_T} = \frac{\frac{K_{a1}K_{a2}}{[\mathrm{H^+}]^2}}{1 + \frac{K_{a1}}{[\mathrm{H^+}]} + \frac{K_{a1}K_{a2}}{[\mathrm{H^+}]^2}}.$$

Equivalently, using $[\mathrm{H^+}] = 10^{-\mathrm{pH}}$,

$$f_0 = \frac{1}{1 + K_{a1}10^{\mathrm{pH}} + K_{a1}K_{a2}10^{2\mathrm{pH}}},$$

$$f_1 = \frac{K_{a1}10^{\mathrm{pH}}}{1 + K_{a1}10^{\mathrm{pH}} + K_{a1}K_{a2}10^{2\mathrm{pH}}},$$

$$f_2 = \frac{K_{a1} K_{a2} 10^{2\mathrm{pH}}}{1 + K_{a1} 10^{\mathrm{pH}} + K_{a1} K_{a2} 10^{2\mathrm{pH}}}.$$
